# Predictability of Macrosomic Birth based on Maternal Factors and Fetal Aneuploidy Screening Biochemical Markers in Hyperglycemic Mothers

**DOI:** 10.7150/ijms.49857

**Published:** 2021-05-13

**Authors:** Junguk Hur, Jinho Yoo, Dayeon Shin, Kwang-Hyun Baek, Sunwha Park, Kyung Ju Lee

**Affiliations:** 1 Department of Biomedical Sciences, University of North Dakota, Grand Forks, North Dakota, USA.; 2 YooJin BioSoft Co., Ltd., Goyang, Gyeonggi-do 10403, Korea.; 3 Department of Food and Nutrition, Inha University, Incheon 22212, Korea.; 4 Department of Biomedical Science, CHA University, Seongnam-Si, Gyeonggi-Do 13488, Korea.; 5 Department of Obstetrics and Gynecology, College of Medicine, Ewha Womans University, 1071 Anyangcheon-ro, Yangcheon-gu, Seoul 07985, Korea.; 6 Department of Obstetrics and Gynecology, Korea University Medicine, Seoul 02841, Korea.; 7 Department of Public Health, Korea University Graduate School, Seoul 02841, Korea.

**Keywords:** maternal biomarker, macrosomic births, maternal hyperglycemia, nomogram

## Abstract

**Background:** Macrosomic birth weight has been implicated as a significant risk factor for developing various adult metabolic diseases such as diabetes mellitus and coronary heart diseases; it has also been associated with higher incidences of complicated births. This study aimed to examine the predictability of macrosomic births in hyperglycemic pregnant women using maternal clinical characteristics and serum biomarkers of aneuploidy screening performed in the first half of pregnancy.

**Methods:** A retrospective observational study was performed on a cohort of 1,668 pregnant women who 1) had positive outcomes after undergoing 50-g oral glucose challenge test (OGCT) at two university-based hospitals and 2) underwent any one of the following maternal biomarker screening tests for fetal aneuploidy: triple test, quadruple test, and integrated test. Logistic regression-based models for predicting macrosomic births using maternal characteristics and serum biomarkers were developed and evaluated for prediction power. A nomogram, which is a graphical display of the best predictable model, was then generated.

**Results:** The study cohort included 157 macrosomic birth cases defined as birth weight ≥3,820 g, which was equivalent to the top 10 percentile of the modeling cohort. Three primary models solely based on serum biomarkers achieved area under curves (AUCs) of 0.55-0.62. Expanded models, including maternal demographic and clinical factors, demonstrated an improved performance by 25% (AUCs, 0.69-0.73).

**Conclusion:** Our prediction models will help to identify pregnancies with an elevated risk of macrosomic births in hyperglycemic mothers using maternal clinical factors and serum markers from routine antenatal screening tests. Prediction of macrosomic birth at mid-pregnancy may allow customized antenatal care to reduce the risk of macrosomic births.

## Introduction

The prevalence of gestational diabetes mellitus (GDM) is steadily increasing worldwide 1, 2. GDM, defined as any degree of glucose intolerance of variable severity with onset or first recognition occurring during pregnancy 3, is a common complication of pregnancy and affects 5%-9% of pregnant women 3, 4. Pre-pregnancy body mass index (BMI) is a known significant risk factor of GDM, and it is highly associated with several adverse pregnancy outcomes, including large for gestational age (LGA), preeclampsia, and cesarean delivery 1, 5. In developing countries, the prevalence of diabetes and obesity in women of reproductive age has rapidly increased over the past decades, and a parallel increase in macrosomia is also expected.

A typical pregnancy is physiologically characterized by weight gain and insulin resistance, with 50%-70% decreased insulin sensitivity in pregnant women compared with that in non-pregnant women 6, 7. However, severe maternal hyperglycemia significantly contributes to abnormal fetal hyperinsulinemia and overgrowth, resulting in LGA and macrosomia. The prevalence of macrosomia in developed countries is between 5% and 20%; however, an increase of 15%-25% has been reported in the past two to three decades. This has mainly been driven by an increase in maternal diabetes, increased gestational BMI, and higher parity 4, 8. Macrosomic birth weight has been implicated as a significant risk factor for developing various adult metabolic diseases such as diabetes mellitus and coronary heart diseases 9-11; it is also associated with higher incidences of complicated delivery such as perinatal asphyxia, shoulder dystocia, cesarean section, prolonged labor, abnormal hemorrhage, perineal trauma, and death 12, 13.

Predicting birth weight is an obstetrically important but difficult challenge. A reference birth weight curve has been constructed based on birth weights per gestational age (GA) 14. However, a birth weight curve cannot predict the exact size; it can just assume that the birth weight is above the 10^th^ percentile 15. Additionally, growth velocity might be associated with perinatal morbidity independent of birth weight, especially with diminished growth or excessive fetal growth. A few available risk prediction models have been developed to assist the decision-making process regarding the management of macrosomia. Fetal overgrowth during pregnancy has been measured using only obstetrical ultrasonography based on fetal structural dimensions within one week prior to delivery. Quantitative assessments using various maternal factors to accurately predict term birth weight have also been developed for evaluation near term.

Some studies have reported the potential usability of maternal biomarkers from fetal aneuploidy screening tests in predicting adverse pregnancy outcomes 16, 17. Particularly, one such biomarker estrogen produced in the placenta has been suggested to have a normal endocrine effect during pregnancy and maternal estrogen levels at delivery were found to be significantly and positively correlated with neonatal birth weight 18, 19. Our previous study 20 also showed that high levels of unconjugated estriol in the maternal serum during the early second trimester of pregnancy are a useful predictor of gestational diabetes development through routinely measurement results of early second-trimester biochemical marker for fetal aneuploidy 21. Considering the availability of these biomarkers, a predictive model using these biomarkers would be useful in clinics to detect macrosomic births earlier than the term pregnancy.

Therefore, we investigated the first- and second-trimester maternal biomarkers for fetal aneuploidy as well as the maternal clinical factors for their predictability of macrosomic birth hyperglycemic pregnant women. For clinical application, we used a combination of fetal and maternal data available at two antenatal visits and developed a graphical display of the best predictable model of macrosomic births.

## Methods

### Study participants

This study was a retrospective observational study. The data were obtained from pregnant women who delivered between July 1, 2007 and December 31, 2015 at two university-based hospitals in Korea, Kangnam CHA Medical Centre and Ewha Womans University Mokdong Hospital. The participants were pregnant women who had positive outcomes of 1-hour 50-g OGCT, which is equivalent to a glucose level >140 mg/dL at around 24-28 weeks' gestation. Participants were excluded if they were missing any of the early pregnancy aneuploidy screening test results and had twin pregnancy, fetal anomaly, hypertensive disorder before pregnancy, pre-existing diabetes, and missing pre-pregnancy or delivery weights. GDM was defined as two or more positive results in a 3-hour 100-g oral glucose tolerance test (OGTT): fasting ≥95 mg/dL, 1 hour ≥180 mg/dL, 2 hours ≥155 mg/dL, and 3 hours ≥140 mg/dL, or one or more positive results in a 2-hour 75-g oral glucose tolerance test (OGTT): fasting ≥92 mg/dL, 1 hour ≥180 mg/dL, and 2 hours ≥153 mg/dL. Based on the delivery date, the eligible subjects were divided into two groups: training set (delivery date on or before December 31, 2009) and testing set (delivery date on or after January 1, 2010).

### Variables

All participants underwent either of the maternal biomarker screening tests for fetal aneuploidy: triple test, quadruple test, or integrated test, comprising of pregnancy-associated plasma protein-A (PAPP-A), alpha-fetoprotein (AFP), free beta-human chorionic gonadotropin (hCG), unconjugated estriol (uE3), and inhibin A. Biochemical indices at sampling were adjusted for maternal weight and GA and reported as multiple of the median (MoM) values of these parameters. Study participants' demographic characteristics and risk factors, including age, pre-pregnancy BMI, parity, systolic/diastolic blood pressure (SBP/DBP), glucose, and lipid levels were obtained during their clinic visit for 50-g OGTT. GA in days was measured from the first day of the last menstrual period. If uncertain or the last menstrual period was unknown, GA was determined using sonography.

### Macrosomic birth

Typical macrosomia is defined as a birth weight of ≥4,000 g 8, whereas LGA is defined as birth weight ≥90 percentiles based on GA, as first introduced in Williams et al.'s fetal growth table 22. In this study, instead of using the “typical” macrosomia, we defined “macrosomic birth” as birth weight ≥90 percentiles (3,820 g) of our modeling dataset, irrespective of GA.

### Construction of prediction models and internal validation

Binary logistic regression analysis was performed to analyze the effects of each potential predictor of macrosomic birth. For constructing best-fit prediction models, multivariable binary logistic regression analysis was performed using a backward stepwise procedure as a variable selection method to minimize Akaike information criterion. Three primary models were constructed using sets of biomarkers (PAPP-A, AFP, hCG, uE3, and inhibin A), routine prenatal triple, quadruple, and integrated screen tests. The primary models were expanded with significant demographic and clinical factors from the univariate analysis.

The discrimination power and calibration power of the constructed models were estimated using area under the curve (AUC) and Hosmer-Lemeshow test, respectively. For internal validation, leave-one-out cross-validation was performed to estimate the reliability of the constructed model. Receiver operating characteristic (ROC) curve analysis was performed to analyze potential variables to predict macrosomic birth. The cut-off values were selected to maximize the sum of sensitivity and specificity, which were used to transform potential variables to binary predictors. The prediction performance was compared among the constructed models using net reclassification improvement (NRI) and integrated discrimination improvement (IDI) analyses. For the practical application of the prediction model in clinical settings, we also developed a nomogram, which is a graphical display of the best performing model for the prediction of macrosomic births.

### Software and basic statistics

R language version 3.3.3 (R Foundation for Statistical Computing, Vienna, Austria), T&F program ver. 2.9 (YooJin BioSoft, Goyang, Korea), and IBM SPSS Statistics for Windows, Version 22 (IBM Corp., Armonk, New York, USA) were used for all statistical analyses and prediction modeling. Data are expressed as mean ± standard deviation for continuous variables. When variables were normally distributed, we performed a mean difference test between two-sample groups defined by macrosomia using a Student's t-test or Welch's t-test as appropriate. For non-normally-distributed variables, the Mann-Whitney U test was used. For categorical variables, data are expressed as simple number and percentage, *N* (%). Chi-square test or Fisher's exact test was performed using a contingency table to assess the association between macrosomic birth and other categorical variables as appropriate.

### Ethics statement

The Institutional Review Boards of CHA Kangnam Medical Centre (IRB No: KNC 10-025) and Ewha Womans University Mokdong Hospital (IRB No: 2020-01-012) approved the protocol of this study.

## Results

### Characteristics of the study participants

**Figure 1:**
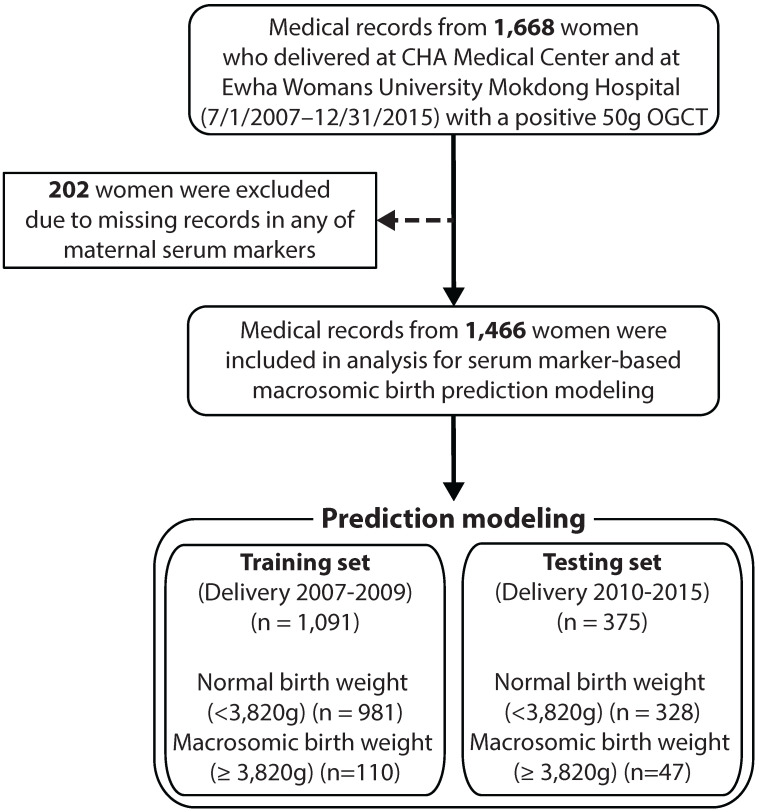
** Flow diagram for study participants.** OGCT: oral glucose challenge test.

Figure 1 illustrates the flow diagram of the study participants. Initially, we collected data from 1,668 women who delivered with a record of positive 50-g OGCT between July 1, 2007 and December 31, 2015 at Kangnam CHA Medical Centre and Ewha Womans University Mokdong Hospital. Subjects were excluded if they had any missing data in any of the maternal serum markers, resulting in a total of 1,466 subjects. Participants were divided into two groups based on birth weight: normal with a birth weight <90^th^ percentile (3,820 g) and macrosomic birth with a birth weight ≥90^th^ percentile.

Table 1 summarizes the demographic and clinical characteristics of the study subjects, who were included in the building prediction models. The ages of the normal and macrosomic birth groups were 32.89 ± 3.90 and 33.32 ± 3.54 years, respectively, which were not significantly different. Higher parity was more likely in the macrosomic birth group than the normal group; however, the difference was not significant. Pre-pregnancy BMI was significantly higher in the macrosomic birth group (22.71 ± 3.73 kg/m^2^) than in the normal group (21.02 ± 2.89 kg/m^2^). Obesity was also significantly associated with macrosomic birth, with approximately three times more obese subjects in the macrosomic birth group (25.5*%*) than in the normal group (9.0%). The levels of biomarkers (PAPP-A, AFP, uE3, hCG, inhibin A, white blood cell [WBC], hemoglobin [Hb], cholesterol, and glucose levels) and apart from weight gain, other clinical factors (nuchal translucency, systolic blood pressure, and diastolic blood pressure) were not significantly different between the two groups until they underwent 50-g OGCT. GDM was significantly associated with macrosomic birth (*P*=0.039). The demographic and clinical characteristics of the subjects used for model performance evaluation are available in Table S1.

Variables were selected as potential predictors in building macrosomic birth predictive models (Tables S2 and S3): six aneuploidy blood marker variables (AFP, hCG, uE3, inhibin A, PAPP-A, and NT), eight other continuous variables (age, WBC, SBP, DBP, Hb, cholesterol, glucose, and weight gain until 50-g OGCT), and five categorical variables (family history of diabetes, family history of hypertension, obesity group by pre-pregnancy BMI, GDM group, and parity from these tests). None of the variables that are only available at delivery was included in the models. Among the included variables, only obesity, GDM groups, and uE3 levels were statistically significant with respect to macrosomic birth (Table S4).

### Macrosomic birth prediction models using multiple maternal serum indices

The models were evaluated for their prediction performance on a testing set, consisting of 328 normal birth and 47 macrosomic birth cases. Three primary models, which were constructed using sets of biomarkers (M1: triple screen test set; M2: quadruple screen test set; M3: integrated screen test set), achieved marginally significant discrimination ability (Figure S1). The M3 model with five biomarkers demonstrated the highest performance (AUC=0.62) among the primary models (Table 2).

### Refinement of prediction models using demographic and clinical factors

**Figure 2:**
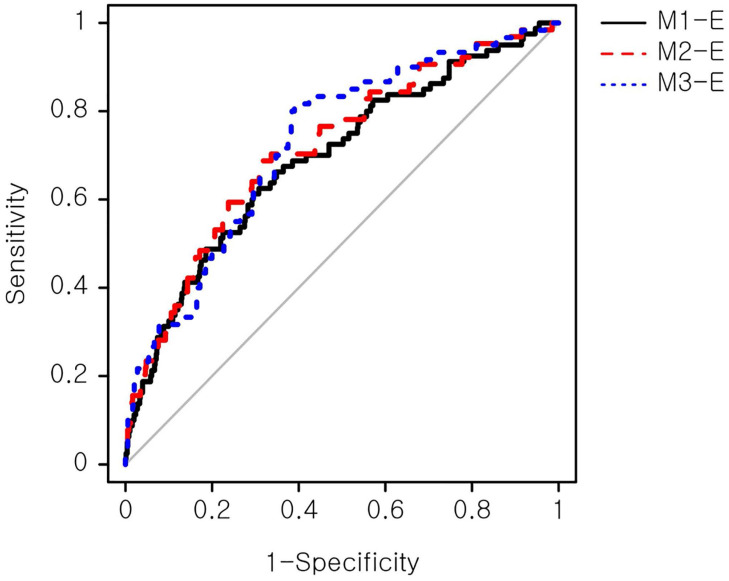
** ROC curve of the expanded prediction models**. The prediction performance of the expanded models was evaluated. Sensitivity, also known as true positive rate, was calculated as (true positive)/(true positive + false positive). Specificity, also known as true negative rate, was calculated as (true negative)/(false negative + true negative). ROC: receiver operating characteristic.

The biomarker-based models were refined with maternal demographic and clinical factors that showed a significant association with the macrosomic birth group in Table 1. These additional factors were obesity, hemoblogin, and weight gain until 50-g OGCT. Three expanded models (Table 3), namely M1-E, M2-E, and M3-E, demonstrated improved prediction performance for macrosomic birth compared with the primary models by a maximum of 25%, achieving AUCs of 0.69-0.73 compared with 0.55-0.62 of the primary models. The M3-E achieved the best performance (AUC=0.73), suggesting a 73% chance that the model could distinguish between normal and macrosomic birth classes. A receiver operating characteristic (ROC) curve, illustrating the trade-off between sensitivity and specificity, was generated to visualize the classification performance (Figure 2). However, no significant performance difference was observed among the three models according to NRI and IDI analyses (Table S5). An additional model only with the above clinical factors, named M-Env, was created. This M-Env model achieved an AUC value of 0.70, slightly lower than that of integrative M3-E; however, the difference was not significantly different according to DeLong's test comparing the two AUCs (*P*-value = 0.249). Although the overall AUC difference was not significant, the addition of serum markers significantly improved the sensitivity and specificity of our integrative model (M3-E). Both NRI and IDI demonstrated that M3-E significantly improved specificity and sensitivity compared to M-Env (Table S6).

### Nomogram

**Figure 3:**
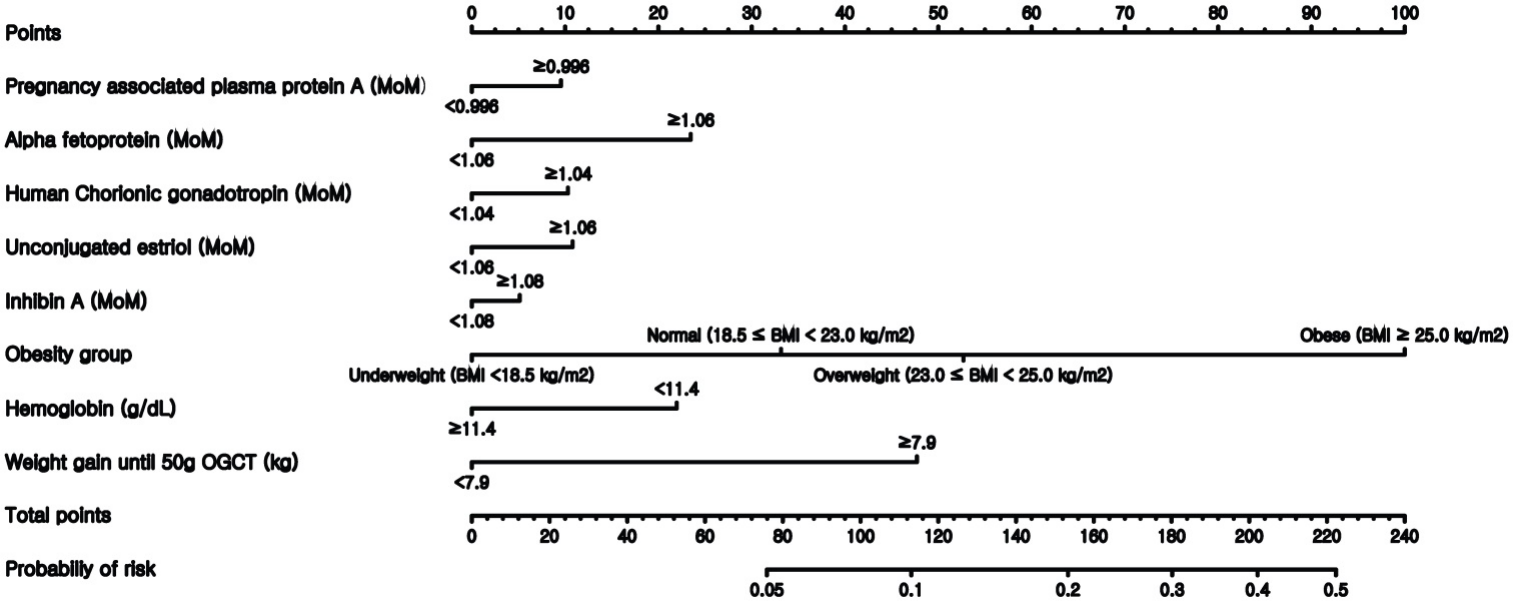
** Nomogram to predict the probability of macrosomic birth.** This nomogram was generated based on the best performing expanded model M3-E.

Finally, nomograms using the variables included in the expanded models were constructed after converting all numeric continuous variables into binary variables. Figure 3 illustrates the nomogram based on the M3-E model, which includes AFP, hCG, estriol, inhibin, obesity group, Hb, and weight gain before 50-g OGTT. Examples of the nomogram's predictive capability are illustrated by calculating macrosomic birth at the midpoint of pregnancy.

## Discussion

This study demonstrated that the prediction of macrosomic birth is possible before the second half of pregnancy or around the time when the OGCT is performed. This was done using a combination of biomarkers from the fetal aneuploidy screening test and maternal demographic characteristics, including biochemical indices that are routinely measured during the first- and second-trimester screening tests for chromosomal abnormalities in hyperglycemic pregnant women.

Our study provides further evidence that macrosomic fetal growth may be predetermined by maternal and fetal parameters already identifiable in the first half of pregnancy. So far, obstetricians have relied on estimated body weight with fetal biometry using sonography to counsel a woman about having a macrosomic birth. Because the sonography is performed right before the end of pregnancy, it has not been possible for clinicians to detect pregnancies with a high risk of macrosomic birth, which is an adverse pregnancy outcome; they have also been unable to intervene at the earlier stages of pregnancy. Our nomogram includes a significant modifiable maternal factor, which is maternal weight gain up to the time of undergoing 50-g OGTT. Therefore, the prediction of a high risk of macrosomic birth can be used to recommend lifestyle changes to women during pregnancy. The earlier the risk prediction is performed, the better the chances of successful risk management during pregnancy.

The benefits of early intervention during pregnancy are well represented in the Barker hypothesis 23. According to the hypothesis, the health of newborns is heavily affected by various maternal conditions, including but not limited to nutrition, maternal obesity, and GDM. The fetus develops rapidly at the later stages of pregnancy. Therefore, the early detection of macrosomia at an early stage will enable an intervention during or after mid-pregnancy via lifestyle education or instructions such as eating habits and physical activities for optimization of healthy weight gain in pregnant women. Though limited, lifestyle interventions in early pregnancy have been shown to be beneficial in preventing GDM 24, 25.

The current study attempted to predict birth weight using factors obtained in the first half of pregnancy. Our predictive models based only on the sets of maternal biomarkers used in the triple, quadruple, and integrated fetal aneuploidy screening tests demonstrated positive predictive performance. The inclusion of maternal characteristics obtained when the OGCT was performed during mid-pregnancy significantly improved the performance of these models. When we built and evaluated the M3-E model on the complete data set, the same performance was achieved with an AUC value of 0.73 (data not shown). This suggests that the model developed using relatively old data (2007-2009) would still be valid for applying to more recent data.

The prediction performance of our model is comparable to others' models. One study used maternal serum markers of the 11-14 week screening and sonogram-based fetal size measurement to predict LGA cases 26. The study reported that hyperglycemia was a causal factor of LGA, and their predictive model for LGA achieved an AUC of 0.6901 (p<0.0001) 26. Our predictive model achieved better predictive performance without requiring a sonogram; hence, it has better potential to be clinically applied to prevent poor pregnancy outcomes.

Pregnancies often result in adverse outcomes, and it is crucial to identify pregnancies at an elevated risk of developing adverse outcomes as early as possible. We previously reported that uE3, one of the pregnancy blood biomarkers, was highly associated with the development of GDM but not with macrosomia in a cohort from Korea 27. However, in this study, uE3 was found to be the most significant factor for building a logistic regression-based predictive model of macrosomic birth using maternal serum markers that are routinely measured during early or mid-pregnancy. This discrepancy can be attributed to the different birth weight cut-offs for defining macrosomic birth in this study (fetal birth weight of ≥3,820 g equivalent to the 90^th^ percentile of hyperglycemic mothers) compared with other studies (fetal birth weight of ≥4,000 g in the general population). A cohort-specific cut-off was used in this study because different levels of metabolic risks are observed at the same BMI across different ethnic groups 28, and different ranges of BMI for defining obesity are recommended for different ethnicities 29, 30.

Although no direct association was noted between macrosomia and uE3 level, a recent study of Chinese women reported significantly lower uE3 and AFP levels in women with GDM than in women without pregnancy-related complications; there was also a significantly over-represented proportion of macrosomia (14.29% of the GDM group) and LGA (25.82% of the GDM group) 31. Our previous study also reported a statistically significant association between uE3 and GDM (OR=0.41; 95% CI 1.85-9.11) and positive trends with LGA (OR=2.35; 95% CI 0.69-7.96) and macrosomia (OR=2.76; 95% CI 0.81-9.41) 20. These results suggest that uE3, GDM, and fetal growth are all closely associated.

One possible mechanism is the decreased insulin sensitivities in pregnant women, which would contribute to the development of GDM 6, 7. Compared with non-pregnant women, typical pregnant women have 50-70% lower insulin sensitivity. Although the role of the maternal hormone estrogen during the pregnancy adaptation process is largely unknown, E2 (estradiol also known as 17β-estradiol) has been shown to directly act on beta-cells of the pancreas to promote insulin synthesis and beta-cell survival 32. Maternal progesterone and estrogen levels constantly increase throughout pregnancy until delivery 33. Progesterone and E2 are secreted by the corpus luteum during early pregnancy, and the developed placenta continues to secrete these hormones and E3 (which shows much higher levels than E2 in the serum) throughout normal pregnancy 33. E3 has been shown to directly induce insulin resistance in adipocytes in cultures, possibly by reducing insulin-simulated glucose transport 34. However, the detailed mechanisms are yet to be explored.

There are multiple strengths in our study and the developed models. Our models primarily rely on biomarkers collected during routine aneuploidy tests; thus, there is no need to perform any additional tests. Other clinical factors used in the models are also routinely obtained during typical prenatal evaluations. Additionally, the nomograms developed in this study will allow the early estimation of macrosomic births by clinicians and consequently provide early interventions.

This study had a few limitations. First, the study cohort was obtained from two hospitals between 2007 and 2015 in Korea; therefore, there could be a selection bias that may not represent all pregnant women. The study cohort is relatively small, and the data used were not nationwide (*n*=1,468 with approximately 10% cases of macrosomic births), requiring caution in interpreting the results. However, recent studies have shown significant increases in obesity and GDM, but no statistically significant increases in the incidence of macrosomia and LGA have been observed in longitudinal studies 35-37. Second, the blood biomarker data were reported as MoM levels and adjusted for maternal age and weight, which may vary with respect to geographic region, ethnicity, and analytic method. Third, the individual points in the nomogram, contributing to the final score, may not completely represent the actual magnitude of the association between the patient characteristic and macrosomic birth. Because the range of points is limited, patients with very different risks may still appear to have the same probability of developing macrosomic birth. Finally, our models were not applicable during early pregnancy because they relied on markers available only during mid-pregnancy when fetal aneuploidy screenings and OGCT were performed. Further research is warranted to identify other biomarkers; this would improve the prediction performance and enable the development of other predictive models using only markers from early pregnancy, thereby allowing interventions as early as possible.

In conclusion, our prediction models of macrosomic birth may help to identify a substantial proportion of hyperglycemic mothers with a high risk of developing macrosomic birth using early second-trimester routine screening biomarkers for chromosomal aneuploidy without requiring additional tests. Although our models cannot completely predict macrosomic births, the models and our nomograms may be useful for customizing antenatal care to reduce the risk of developing macrosomic births.

## Supplementary Material

Supplementary figure and tables.

## Figures and Tables

**Table 1:** Demographic and clinical characteristics of the study subjects

Variable	Normal	Macrosomic birth (90%)	*P* value
Number of subjects (%)	981 (89.9)	110 (10.1)	
Age (years)	32.88 ± 3.89	33.32 ± 3.54	0.188
**Family history of diabetes mellitus**			0.955
No	787 (80.2)	88 (80.0)	
Yes	194 (19.8)	22 (20.0)	
**Family history of hypertension**			0.982
No	775 (79.0)	87 (79.1)	
Yes	206 (21.0)	23 (20.9)	
Pre-pregnancy BMI (kg/m^2^)	21.02 ± 2.89	22.71 ± 3.73	< 0.001**
**Parity**			0.075
0	651 (66.4)	72 (65.5)	
1	262 (26.7)	25 (22.7)	
2	62 (6.3)	10 (9.1)	
3	5 (0.5)	3 (2.7)	
4	1 (0.1)	0 (0.0)	
**Obesity**			< 0.001**
Normal (18.5 ≤ BMI < 23.0 kg/m^2^)	602 (61.4)	61 (55.5)	
Underweight (BMI <18.5 kg/m^2^)	173 (17.6)	8 (7.3)	
Overweight (23.0 ≤ BMI < 25.0 kg/m^2^)	118 (12.0)	13 (11.8)	
Obese (BMI ≥25.0 kg/m^2^)	88 (9.0)	28 (25.5)	
**GDM group**			0.039*
Normal	746 (76.4)	72 (67.3)	
GDM	231 (23.6)	35 (32.7)	
**Gestational age (weeks)**			
First-trimester screening	11.91 ± 0.68	11.91 ± 0.67	0.848
Second-trimester screening	16.37 ± 0.76	16.29 ± 0.64	0.378
50g OGCT	26.81 ± 1.47	26.8 ± 1.51	0.871
Delivery	38.92 ± 1.51	39.76 ± 1.02	< 0.001**
Nuchal translucency (cm)^ $^	0.12 (0.10 - 0.15)	0.14 (0.10 - 0.16)	0.129
Pregnancy associated plasma protein A (MoM)^ $^	1.00 (0.63 - 1.56)	1.02 (0.54 - 1.8)	0.768
Alpha fetoprotein (MoM)	1.10 ± 0.36	1.12 ± 0.33	0.461
Unconjugated estriol (MoM)	1.08 ± 0.33	1.20 ± 0.49	0.095
Human Chorionic gonadotropin (MoM)^ $^	1.04 (0.74 - 1.40)	1.03 (0.80 - 1.36)	0.969
Inhibin A (MoM)^ $^	1.09 (0.83 - 1.43)	1.08 (0.86 - 1.48)	0.721
Systolic blood pressure (mmHg)	113.69 ± 11.98	114.91 ± 10.66	0.231
Diastolic blood pressure (mmHg)	67.48 ± 8.29	67.09 ± 7.45	0.707
White blood cells (count/mL)	9213.56 ± 1936.17	9617.8 ± 2276.92	0.082
Hemoglobin (g/dL)	11.38 ± 0.9	11.28 ± 0.82	0.212
Total cholesterol (mg/dL)	234.08 ± 39.76	229.63 ± 39.08	0.201
Glucose (mg/dL)^ $^	152 (145 - 164)	154 (145 - 166)	0.268
Weight gain until 50g OGCT (kg)	7.89 ± 3.74	8.60 ± 3.61	0.016*

Statistical significance was calculated using T-test, Mann-Whitney U test^$^, or Fisher's exact test depending on the data type. Continuous variables are expressed as mean ± standard deviation or median with inter-quartile range^$^, considering skewness of the data distribution. *: *P*<0.05; **: *P*<0.001; BMI: body mass index; MoM: multiple of the median; OGCT: oral glucose challenge test.

**Table 2:** Comparison of area under curve among the three primary prediction models

Predictor	AUC (95% CIs)	*P* value	Sensitivity	Specificity	Cut-off	*P* value for AUC comparison
M1	0.55 (0.49, 0.62)	0.097	0.701	0.431	0.085	Reference
M2	0.61 (0.54, 0.68)	0.003	0.754	0.43	0.078	0.171
M3	0.62 (0.54, 0.69)	0.002	0.698	0.514	0.087	0.235

Cut-off was selected to maximize the sum of sensitivity and specificity. AUC: area under curve; CIs: confidence intervals; M1: prediction model consisting of Alpha fetoprotein (MoM), Human Chorionic gonadotropin (MoM), and Unconjugated estriol (MoM); M2: prediction model consisting of M1 + Inhibin A (MoM); M3: prediction model consisting of M2 + Pregnancy associated plasma protein A (MoM). *P* value for AUC comparison was computed using DeLong's test.

**Table 3:** Expanded prediction models for macrosomic birth

Predictor	AUC (95% CIs)	*P* value	Sensitivity	Specificity	Cut-off	*P* value for AUC comparison
M1-E	0.69 (0.63, 0.76)	< 0.001	0.612	0.705	0.100	Reference
M2-E	0.72 (0.65, 0.78)	< 0.001	0.688	0.688	0.094	0.495
M3-E	0.73 (0.66, 0.79)	< 0.001	0.817	0.610	0.087	0.266

Cut-off was selected to maximize the sum of sensitivity and specificity. AUC: area under curve; CIs: confidence intervals; M1-E: prediction model consisting of M1, Obesity group, Hemoglobin (g/dL), and Weight gain until 50g OGCT (kg); M2-E: prediction model consisting of M2, Obesity group, Hemoglobin (g/dL), and Weight gain until 50g OGCT (kg); M3-E: prediction model consisting of M3, Obesity group, Hemoglobin (g/dL), and Weight gain until 50g OGCT (kg). *P* value for AUC comparison was computed using DeLong's test.
