# Supplementary material for: Predictability of Macrosomic Birth based on Maternal Factors and Fetal Aneuploidy Screening Biochemical Markers in Hyperglycemic Mothers
Source: Int J Med Sci. 2021 May 13;18(12):2653–60. doi: 10.7150/ijms.49857 (PMC8176187; doi:10.7150/ijms.49857)

Table S1. Demographic and clinical characteristics of the study subjects (testing cohort)

**Variable**	**Normal (Birth weight <3,820g)**	**Macrosomic birth (Birth weight: ≥3,820g)**	**P-value**
Sample No (%)	328 (87.47)	47 (12.53)	
Age (year)	34.74 ± 3.68	34.34 ± 4.20	0.542
Family history of Diabetes			0.008**
No	219 (66.8)	22 (46.8)	
Yes	109 (33.2)	25 (53.2)	
Family history of Hypertension			0.804
No	236 (72.0)	33 (70.2)	
Yes	92 (28.0)	14 (29.8)	
Pre-pregnancy BMI (kg/m2)	22.94 ± 4.06	25.51 ± 4.78	0.001**
Parity			0.394
0	193 (59.2)	22 (46.8)	
1	107 (32.8)	20 (42.6)	
2	23 (7.1)	4 (8.5)	
3	1 (0.3)	1 (2.1)	
4	1 (0.3)	0 (0.0)	
6	1 (0.3)	0 (0.0)	
Obesity group			0.003**
Normal (18.5 ≤ BMI < 23.0 kg/m2)	149 (45.8)	14 (31.1)	
Underweight (BMI <18.5 kg/m2)	33 (10.2)	0 (0.0)	
Overweight (23.0 ≤ BMI < 25.0 kg/m2)	61 (18.8)	10 (22.2)	

Obese (BMI ≥ 25.0 kg/m2)	82 (25.2)	21 (46.7)	
GDM group			0.474
Normal	160 (56.1)	19 (50)	
GDM	125 (43.9)	19 (50)	
Gestational age (weeks)			
First-trimester screening	11.91 ± 0.74	11.82 ± 0.64	0.472
Second-trimester screening [$]	16.29 (15.71 - 16.71)	16.29 (15.86 - 16.57)	0.696
50g OGCT	26.33 ± 1.91	26.53 ± 2.24	0.601
Delivery [$]	38.57 (37.43 - 39.29)	38.86 (38.14 - 39.57)	0.024*
Nuchal translucency (cm)	1.19 ± 0.44	1.19 ± 0.5	0.936
Pregnancy associated plasma protein A (MoM)	1.04 ± 0.65	1.10 ± 0.47	0.549
Alpha fetoprotein (MoM)	1.09 ± 0.43	1.00 ± 0.40	0.306
Unconjugated estriol (MoM)	0.97 ± 0.26	1.00 ± 0.23	0.548
Human Chorionic gonadotropin (MoM) [$]	1.02 (0.76 - 1.35)	1.23 (0.81 - 1.74)	0.128
Inhibin A (MoM) [$]	1.00 (0.78 - 1.33)	1.10 (0.81 - 1.52)	0.365
Systolic blood pressure (mmHg)	117.1 ± 15.58	120.03 ± 14.04	0.238
Diastolic blood pressure (mmHg)	69.8 ± 10.65	71.49 ± 8.57	0.274
White blood cells (count/mL)	9561.8 ± 2355.85	9479.62 ± 1618.39	0.817
Hemoglobin (g/dL)	11.76 ± 0.98	11.8 ± 0.90	0.841
Total cholesterol (mg/dL)	237 ± 41.83	237.02 ± 50.6	0.998
Glucose (mg/dL)	128.9 ± 33.02	143.14 ± 40.32	0.077
Weight gain until 50g OGCT (kg) [$]	6.50 (4.03 - 9.17)	6.50 (3.9 - 10.6)	0.698

Continuous variables are expressed as mean ± standard deviation or median (inter-quartile range). Statistical significance was calculated using independent two sample T test, Mann-Whitney U test[$] (considering their skewness), or chi-squared test depending on data type. *: p<0.05; **: p<0.001; BMI: body-mass index; MoM: multiple of the median; OGCT: oral glucose challenge test

Table S2. ROC curve analysis of macrosomic birth for continuous variables.

Predictor	AUC (95% CIs)	P-value	Sensitivity	Specificity	PPV	NPV
Age (year)	0.538 (0.485 - 0.591)	0.189	0.800	0.314	0.104	0.928
Pre-pregnancy BMI (kg/m^2)	0.643 (0.588 - 0.698)	< 0.001	0.591	0.637	0.110	0.933
Nuchal translucency (cm)	0.551 (0.483 - 0.620)	0.130	0.500	0.615	0.125	0.906
Pregnancy associated plasma protein A (MoM)	0.511 (0.430 - 0.593)	0.768	0.365	0.754	0.110	0.919
Alpha fetoprotein (MoM)	0.524 (0.464 - 0.584)	0.461	0.690	0.420	0.104	0.949
Unconjugated estriol (MoM)	0.554 (0.488 - 0.621)	0.095	0.632	0.482	0.100	0.925
Human Chorionic gonadotropin (MoM)	0.501 (0.439 - 0.564)	0.969	0.874	0.169	0.091	0.902
Inhibin A (MoM)	0.513 (0.443 - 0.583)	0.721	0.913	0.145	0.091	0.886
Systolic blood pressure (mmHg)	0.536 (0.478 - 0.593)	0.231	0.433	0.642	0.103	0.911
Diastolic blood pressure (mmHg)	0.511 (0.454 - 0.569)	0.707	0.510	0.533	0.112	0.902
White blood cells (count/mL)	0.553 (0.490 - 0.616)	0.082	0.500	0.632	0.107	0.929
Hemoglobin (g/dL)	0.538 (0.484 - 0.592)	0.212	0.740	0.387	0.099	0.900
Total cholesterol (mg/dL)	0.540 (0.477 - 0.603)	0.201	0.553	0.544	0.103	0.907
Glucose (mg/dL)	0.533 (0.473 - 0.592)	0.269	0.686	0.399	0.105	0.941
Weight gain until 50g OGCT (kg)	0.572 (0.513 - 0.631)	0.016	0.495	0.653	0.105	0.917

AUC: area under curve; BMI: body-mass index; CIs: confidence intervals; MoM: multiple of the median; NPV: negative predictive value; OGCT: oral glucose challenge test; PPV: positive predictive value; ROC: receiver operating characteristic

Table S3. Association analysis between macrosomic birth and categorical variables.

Variable	N (%)	Normal	Macrosomic Birth (90th %)	P-value	OR (95% CIs)
Number of subjects (%)	1091 (100)	981 (89.9)	110 (10.1)		
Age (years)	1091 (100)			0.88	
<35	718 (65.8)	647 (66)	71 (64.5)		1
≥35 ~ <40	317 (29.1)	283 (28.8)	34 (30.9)		1.095 (0.711-1.686)
≥40	56 (5.1)	51 (5.2)	5 (4.5)		0.893 (0.345-2.312)
Family history of diabetes	1091 (100)			0.955	
No	875 (80.2)	787 (80.2)	88 (80)		1
Yes	216 (19.8)	194 (19.8)	22 (20)		1.014 (0.62-1.66)
Family history of hypertension	1091 (100)			0.982	
No	862 (79)	775 (79.0)	87 (79.1)		1
Yes	229 (21)	206 (21.0)	23 (20.9)		0.995 (0.613-1.615)
Obesity group (by pre-pregnancy BMI)	1091 (100)			< 0.001**	
Normal (18.5 ≤ BMI < 23.0 kg/m^2)	663 (60.8)	602 (61.4)	61 (55.5)		1
Underweight (BMI <18.5 kg/m^2)	181 (16.6)	173 (17.6)	8 (7.3)		0.456 (0.214-0.972)
Overweight (23.0 ≤ BMI < 25.0 kg/m^2)	131 (12)	118 (12.0)	13 (11.8)		1.087 (0.579-2.042)
Obese (BMI ≥ 25.0 kg/m^2)	116 (10.6)	88 (9.0)	28 (25.5)		3.14 (1.904-5.178)
GDM group	1084 (100)			0.033*	
Normal	602 (55.5)	555 (56.8)	47 (43.9)		1
Prediabetes	216 (19.9)	191 (19.5)	25 (23.4)		1.546 (0.926-2.58)
GDM	266 (24.5)	231 (23.6)	35 (32.7)		1.789 (1.125-2.845)
Parity	1091 (100)			0.075	
0	723 (66.3)	651 (66.4)	72 (65.5)		1
1	287 (26.3)	262 (26.7)	25 (22.7)		0.863 (0.535-1.39)
2	72 (6.6)	62 (6.3)	10 (9.1)		1.458 (0.716-2.969)
3	8 (0.7)	5 (0.5)	3 (2.7)		5.425 (1.27-23.172)
4	1 (0.1)	1 (0.1)	0 (0.0)		2.995 (0.121-74.212)

White blood cells (counts/mL)	1005 (100)			0.049*	
<14000	984 (97.9)	889 (98.2)	95 (95)		1
≥14000	21 (2.1)	16 (1.8)	5 (5)		2.924 (1.048-8.161)

BMI: body-mass index; GDM: gestational diabetes mellitus; OR (95% CIs): odds ratio with 95% confidence intervals; P-value: computed chi-squared test or Fisher's exact test

Table S4. Univariate logistic regression analysis of 19 variables for the prediction of macrosomic birth.

Predictor	OR (95%CIs)	P-value
Age (years)	1.029 (0.979 - 1.083)	0.261
Family history of diabetes		0.955
Yes vs. No	1.014 (0.62 - 1.66)	0.955
Family history of hypertension		0.982
Yes vs. No	0.995 (0.613 - 1.615)	0.982
Parity	1.166 (1.102 - 1.234)	< 0.001**
1 vs. 0		0.157
≥2 vs. 0	0.863 (0.535 - 1.39)	0.544
Obesity	1.729 (0.91 - 3.282)	0.094
Underweight vs. Normal		< 0.001**
Overweight vs. Normal	0.456 (0.214 - 0.972)	0.042*
Obesity vs. Normal	1.087 (0.579 - 2.042)	0.795
GDM	3.14 (1.904 - 5.178)	< 0.001**
Yes vs. No		0.04*
Nuchal translucency (cm)	1.57 (1.021 - 2.413)	0.04*
Pregnancy associated placenta protein A (MoM)	0.86 (0.219 - 3.381)	0.829
Alpha fetoprotein (MoM)	1.159 (0.91 - 1.477)	0.232
Unconjugated estriol (MoM)	1.176 (0.642 - 2.153)	0.6
Human Chorionic gonadotropin (MoM)	2.282 (1.303 - 3.995)	0.004**

Inhibin A (MoM)	1.011 (0.697 - 1.466)	0.955
Systolic blood pressure (mmHg)	0.948 (0.604 - 1.487)	0.815
Diastolic blood pressure (mmHg)	1.009 (0.992 - 1.026)	0.319
White blood cells (count/mL)	0.994 (0.97 - 1.019)	0.641
Haemoglobin (g/dL)	2.69 (0.989 - 7.314)	0.053
Total cholesterol (mg/dL)	0.884 (0.703 - 1.112)	0.293
Glucose (mg/dL)	0.997 (0.992 - 1.003)	0.301
Weight gain until 50g OGCT (kg)	1.007 (0.997 - 1.016)	0.174

GDM: gestational diabetes mellitus; MoM: multiple of the median; OGCT: oral glucose challenge test; OR (95% CIs): odds ratio with 95% confidence intervals; *: $p<0.05$; **: $p<0.001$;

Table S5. Reclassification improvement of models M2-E and M3-E compared to model M1-E.

**Model**	**Index**	**Mean (95% CIs)**	**P-value**
M2-E	NRI (decile)	-0.040 (-0.114 – 0.034)	0.288
	IDI	-0.001 (-0.004 – 0.002)	0.526
M3-E	NRI (decile)	0.020 (-0.104 – 0.144)	0.752
	IDI	0.006 (-0.004 – 0.016)	0.231

NRI: net reclassification improvement; IDI: integrated discrimination improvement analyses.

Table S6. Reclassification improvement of model M3-E compared to model M-Env.

Model	Index	Mean (95% CIs)	P-value
M3-E	NRI (decile)	0.212 (0.071 - 0.353)	0.003
	IDI	0.022 (0.006 - 0.038)	0.007

NRI: net reclassification improvement; IDI: integrated discrimination improvement analyses.

Figure S1. Receiver Operating Characteristic (ROC) curve of the primary prediction models. The prediction performance of the primary models solely using the serum markers were evaluated, where all three models demonstrate marginally significant and better performances than random (AUC > 0.500). Sensitivity, also known as true positive rate, was calculated as (true positive)/(true positive + false positive). Specificity, also known as true negative rate, was calculated as (true negative)/(false negative + true negative).

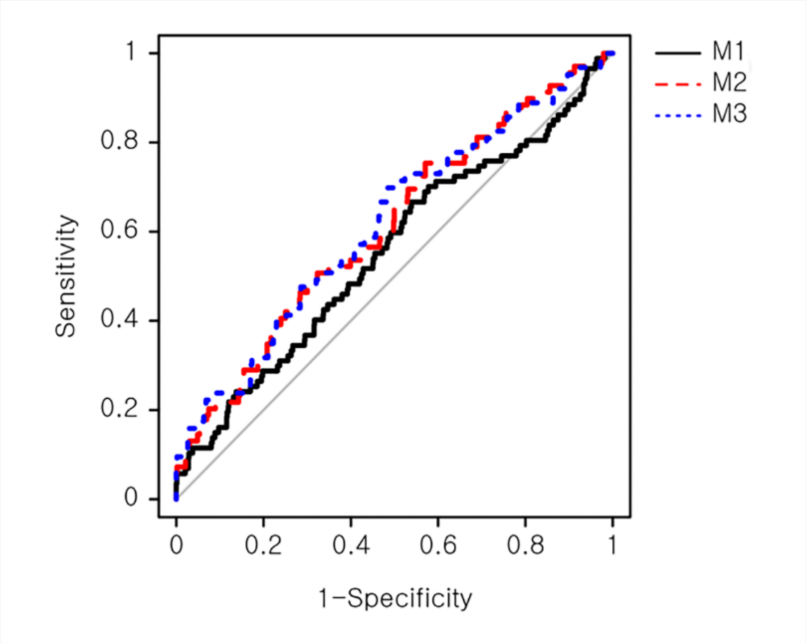

Supplement: Supplementary file 1 — Supplementary figure and tables. [file ijmsv18p2653s1.pdf]
